# We need to talk: clinical competency committees in the key of c(onversation)

**DOI:** 10.1007/s40037-017-0360-2

**Published:** 2017-05-23

**Authors:** Paul A. Hemmer, William F. Kelly

**Affiliations:** Department of Medicine, Uniformed Services University of the Health Sciences, Bethesda, MD USA

James Suroweicki’s book, The Wisdom of Crowds [1], provided examples of how diverse, independently deciding individuals make some decisions and predictions better than any individual, even an expert one. However, there have been epic failures where group dynamics derail decision-making and crowds become herds.

Over the past decades, there has been a steady movement towards competency-based medical education accompanied by assessment programs emphasizing the judgment of experts in promotion and progress decisions [2]. While competency committees are not new in undergraduate medical education [3] or graduate medical education [4], they are now required of all residency training programs in the US accredited by the Accreditation Council for Graduate Medical Education (ACGME) [5] and will soon be required in training programs in Canada [6]. What is not well understood is *how* members of a clinical competency committee (CCC) reach a judgment or conclusion. In their article, Chacine et al. report on their synthesis of literature on group decision-making which yielded three orientations (schema, constructivist, and social influence) that are within a context of ‘moderators’ (moderating influences) that can help to frame an approach to examining* how *CCC decisions are made.

Why is the ‘how’ an important question to answer? Simply put, CCCs represent a visible commitment to the importance of conducting evaluation, reaching judgments, and ensuring fairness to multiple stakeholders – to society, to teachers, and to trainees. Because the work of the CCC is at the very core of the profession of those involved in educational training programs, if we can illuminate and elucidate the elements of how decisions are made, we may develop insight into what makes for highly functioning and effective committees. In short, knowing how a committee arrives at a decision is the first step towards ensuring it is making the *best* one.

## Conversation is the key

Based on our own 30-year experience of placing conversation at the centre of a program of assessment, we believe that the conversation itself holds the key to understanding CCC decisions [3, 7–16]. Conversation ‘is a progression of exchanges’ among participants; each participant changes internally as a consequence of conveying and generating knowledge [17]. It is here that Gordon Pask’s Conversation Theory is relevant [17, 18]. Coming from the field of cybernetics, conversation theory addresses how learning takes place through conversation. At the risk of oversimplification, a series of steps has been summarized as: opening a channel (beginning with a message); commit to engage (engagement on the part of those in the conversation); construct meaning (through an interchange, those involved begin to construct meaning); evolve (participants are in some way changed); converge on agreement (and this includes the ability to ‘teach back’); act or transact (one or more participants agree to do something as a result) [17].

A central feature of conversation theory is ‘reaching agreement over an understanding’ (Converge on Agreement) – in the work of the CCC, this would be the process by which the members reach a decision, consensus, or agreement over the understanding of ‘competence’. The formulation of ‘competence’ might vary depending on the frame of reference (e. g., ACGME, CANMEDS, Entrustable Professional Activities, Reporter-Interpreter-Manager-Educator) but that is the shared understanding which is served by the conversation. While conversation theory would seem most readily applicable to the Constructivist orientation proposed by Chahine et al., it is also relevant to the other orientations. The requirement for the CCC is a recognition of the power of conversation to clarify, calibrate, coordinate, and collaborate. Conversation changes participants in meaningful ways, and understanding how they are changed should be part of the exploration of CCC decision-making.

## How do CCCs reach consensus? It probably depends

The authors recognize that the orientations they propose are not mutually exclusive and that it is probable that all orientations are likely to be used, to one degree or another, in the conversations to reach a consensus about trainees – which orientation predominates (if one does) probably depends on the trainee, the CCC members, and the context. In many ways, this is analogous to competence in patient care that involves factors related to the physician, patient, and encounter [19].

A *competent* competency committee must understand the judgments they make, the feedback an individual trainee needs on their growth to independence, and recognize contextual factors that can impact the group’s decision. Awareness of such ‘moderators’ and decision-making pitfalls is essential and one more benefit of examining how decisions are made.

CCC members, typically program faculty and teachers themselves, are likely going to be ‘meaningfully idiosyncratic’ in how they process information and reach a judgment [20]. While this may lead to voicing differing views, it likely represents more ‘signal’ about the trainee than ‘noise’ from the CCC process.

Resources are likely to be a major ‘moderator’, including financial, adequate faculty training and expertise, and especially time, as longer discussions usually produce better learner feedback. Additionally, the complexity and detail of the competency frameworks themselves (e. g., ACGME, CANMEDS) may also impact decision-making, as could the type, size, and scope of the training program.

Pitfalls of group decision-making include reduced efficiency, a diffusion of responsibility (lack of accountability) which can lead to ‘groupthink’. This is an often unconscious desire for harmony (getting along) or for conformity (deferring to leaders with greater ‘psychological size’). However, prior work from nearly 20 years ago has demonstrated that groupthink or dominance by certain individuals was not evident in CCCs at the undergraduate [3] or graduate [4] level in medical education – what mattered was the information being discussed. Having the most junior person speak first and the committee chair speak last (if at all) may help to limit groupthink or individual dominance. Another scenario, ‘group polarization’ or ‘risk shifting’, is related to this and has occurred when the group’s decision is much more extreme than if made by its individual members. ‘Shared information bias’ occurs when members spend all of their time discussing a portion of information they all have and ignore more important information available to a subset of the members. Use of ‘touchstones’ or ground rules for professional discussions, and reflection and feedback after CCC meetings may improve the process.

## Next steps

In seeking to address the *how* of decision-making by members of a CCC, there may be challenges in identifying CCCs to study, such as whether to try to select examples of effective and less effective committees, how to define that effectiveness (which committees made the ‘best’ decisions), and establishing the trustworthiness of the qualitative information likely to be central to this type of study. Nevertheless, by seeking to understand the *how*, we move forward in our commitment to fairness to society, teachers, and trainees. The authors have suggested a framework to begin the conversation about how CCCs make decisions.
